# Added Value of Whole-Body Diffusion-Weighted Imaging in Patients Undergoing Prostate-Specific Membrane Antigen Positron Emission Tomography

**DOI:** 10.3390/jcm14061833

**Published:** 2025-03-08

**Authors:** Cheng William Hong, Spencer C. Behr, Fei Jiang, Yingbing Wang, Sina Houshmand, Thomas A. Hope

**Affiliations:** 1 Department of Radiology and Biomedical Imaging, University of California San Francisco, San Francisco, CA 94143, USA; spencer.behr@ucsf.edu (S.C.B.); yingbing.wang@ucsf.edu (Y.W.); sina.houshmand@ucsf.edu (S.H.); 2 Helen Diller Comprehensive Cancer Center, University of California San Francisco, San Francisco, CA 94143, USA; 3 Clinical and Translational Science Institute, University of California San Francisco, San Francisco, CA 94143, USA; fei.jiang@ucsf.edu; 4 Department of Radiology, San Francisco VA Medical Center, San Francisco, CA 94121, USA

**Keywords:** prostate cancer, mCRPC, PSMA, diffusion-weighted imaging, WB DWI

## Abstract

**Background/Objectives**: Patients with metastatic castration-resistant prostate cancer (mCRPC) who have Prostate-Specific Membrane Antigen (PSMA)-negative disease have inferior outcomes with radioligand therapy (RLT). The objective of this study is to assess the added value of whole-body (WB) diffusion-weighted imaging (DWI) to PSMA PET for identifying PSMA-negative disease, which is important for risk stratification. **Methods**: Consecutive PSMA PET/MRI exams at our institution, which included WB DWI in patients with mCRPC, were retrospectively reviewed. For both WB DWI and PSMA PET, two independent readers scored 14 anatomic locations, which were considered positive only if both readers identified lesions. The proportion of patients with mismatched disease was summarized descriptively for each anatomic location and overall. The inter-reader agreement was computed with intra-class correlation coefficients (ICCs). **Results**: The study included 41 patients (with a mean age of 71.9 years), and WB DWI identified PSMA-negative lesions in 24% of patients. PSMA PET had higher agreement than DWI, although both had good agreement (ICC: 0.87 and 0.72, respectively). The median overall survival was 442 days in those with mismatched disease vs. 523 days in those without, although this difference is not statistically significant (*p* = 0.49). **Conclusions**: The addition of WB DWI to PSMA PET can identify PSMA-negative disease, which could alter patient management.

## 1. Introduction

Prostate cancer is the most common cancer and the second most common cause of cancer-related death in American men [1]. Metastatic castration-resistant prostate cancer (mCRPC) is a particularly lethal form of prostate cancer, for which there are relatively few treatment options. The VISION study demonstrated that targeting the Prostate-Specific Membrane Antigen (PSMA) using 177Lu-PSMA-617 (vipivotide, Pluvicto, Novartis) improves the quality of life and overall survival in mCRPC, improving the overall survival to 15.3 months compared to 11.3 months with standard of care [2]. The TheraP trial demonstrated improved progression-free survival and higher PSA response rates in patients treated with 177Lu-PSMA-617 compared to cabazitaxel [3].

In the VISION trial, the evaluation of candidates for radioligand therapy (RLT) was based on PSMA positron emission tomography (PET) demonstrating PSMA-avid disease in all measurable sites [4]. In the TheraP trial, which had more stringent eligibility criteria, FDG PET was used in addition to PSMA PET to identify PSMA-negative disease. A total of 18% of patients were excluded because of mismatched FDG-avid disease, resulting in a higher PSA50 response rate (a PSA decrease by >50%) of 66% compared to 44% in the VISION trial [5].

Patients with PSMA-negative disease have inferior outcomes with PSMA RLT, highlighting the importance of identifying PSMA-negative disease for risk stratification [5,6]. A retrospective analysis of patients that would have been screen failures based on the VISION criteria demonstrated a lower PSA50 response rate, shorter PSA progression-free survival, and shorter overall survival, indicating the need for refining patient selection to optimize outcomes [7]. In addition, a secondary analysis of the TheraP trial showed that patients with a high metabolic tumor volume on FDG PET had worse outcomes [5]. Because of these results, it has been suggested that FDG PET should be used to screen patients for RLT. However, the use of FDG PET requires a second imaging exam on a separate day, which is impractical from a workflow, cost, and insurance perspective. FDG PET is, thus, rarely performed in actual practice, despite this clinical need, and a more practical approach is needed.

Diffusion-weighted imaging (DWI) is an MRI sequence where signal intensity is higher for voxels that have non-mobile water molecules (e.g., tumor) compared to voxels that have freely diffusing water molecules. The diffusion sensitizing gradients can be applied with different strengths, characterized by the b-value. Whole-body (WB) DWI is a promising modality for assessing metastases, particularly in bone [8]. It has demonstrated excellent inter- and intra-observer agreements and provides quantitative information [9]. DWI can provide synergistic information as part of a PET/MRI exam in combination with specific non-FDG tracers, potentially detecting lesions that might be negative on PET [10].

We hypothesize that WB DWI can be used to identify PSMA-negative disease, and PSMA PET/MRI could be a practical approach to evaluating candidates for RLT without the need for a second exam [11]. This study assesses the proportion of patients where WB DWI detects PSMA-negative lesions, the locations of PSMA-negative disease detected, and the median survival depending on the presence or absence of mismatched disease. We also assessed the inter-reader agreement of WB DWI and PSMA PET.

## 2. Materials and Methods

### 2.1. Patient Population

This was a single-center retrospective analysis of consecutive clinical PSMA PET/MRI examinations that included WB DWI performed at our institution between January 2022 and October 2022 for the evaluation of patients with mCRPC. This study was approved by the local institutional review board, and individual informed consent was waived as it is a minimal-risk study.

### 2.2. PET/MRI Protocol

PET/MRI examinations were performed on a 3.0T SIGNA PET/MRI scanner (GE Healthcare, Waukesha, WI, USA). Both PET and MRI were performed from vertex to mid-thigh. A total of 35 patients were imaged using ^68^Ga-PSMA-11, and 6 patients were imaged using ^18^F-DCFPyL. For ^68^Ga-PSMA-11, patients received a mean (SD) of 233 (47) MBq (6.3 [1.3] mCi). For ^18^F-DCFPyL, patients received a mean (SD) of 400 (10) MBq (10.8 [0.3] mCi). Image acquisition began with a mean (SD) of 63 (12) minutes after injection. Each exam had 6 bed tasks, and the emission time was 4 min per bed. The PET images were corrected for attenuation, dead time, random events, and scatter, and they were reconstructed using time-of-flight ordered subset expectation maximization (OSEM) using 2 iterations, 28 subsets, and a matrix size of 256 × 256, with a 600 × 250-mm field of view and a slice thickness of 2.8 mm.

For the MRI, axial single-shot fast spin echo (SSFSE), non-contrast fat-suppressed T1-weighted, b = 50, and b = 800 DWI were acquired, and apparent diffusion coefficient (ADC) maps were generated. DWI was acquired using a repetition time (TR) of 4300 ms, an echo time (TE) of 66 ms, and a matrix size of 80 × 128. Other MRI sequences are as previously described [12].

### 2.3. Image Interpretation

DWI was reviewed by two abdominal radiologists blinded to the PSMA PET images. The PSMA PET images were reviewed by two nuclear medicine physicians blinded to the DWI. For each patient, the following anatomic locations were assessed for restricted diffusion on the DWI or PSMA activity on the PSMA PET representing a tumor: lymph nodes (pelvic, retroperitoneal, mediastinal, inguinal, and supraclavicular), bones (pelvic, lumbar, thoracic, cervical spine, upper and lower extremities, and ribs), lung, liver, and others. The other MRI sequences were used for anatomic localization but not for determining if restricted diffusion was present or absent. No specific training or size threshold was provided; the readers were instructed to interpret the images as they would clinically. Each anatomic compartment was considered DWI-positive if both abdominal radiologists rated it as positive. Similarly, it was considered PSMA-positive if both nuclear medicine physicians rated it as positive.

### 2.4. Statistical Analysis

The proportion of patients that had mismatched disease that was identified on the DWI (DWI+/PSMA-) was summarized descriptively for each anatomic location and overall. An inter-reader agreement was computed for the presence or absence of disease using intra-class correlation coefficients (ICCs). ICCs < 0.5 were considered as poor reliability; ICCs between 0.5 and 0.75 were considered as moderate reliability, and ICCs between 0.75 and 0.9 were considered as good reliability. ICCs greater than 0.90 were considered as excellent reliability [13]. The overall survival was compared between patients with non-nodal PSMA-negative disease that was identified on WB DWI vs. those without using a log-rank test, and a Kaplan–Meier curve and the median survival time were computed for both groups.

## 3. Results

A total of 41 patients were included in the analysis. The average age was 71.9 ± 8.9 years, and the median PSA was 80.9 ng/mL (IQR: 40.6–487.0). All patients had metastatic castration-resistant prostate cancer. A total of 86% of patients had a Gleason score of 7 or higher, and 63% of patients had a Gleason score of 8 or higher based on prostate biopsy or prostatectomy (Table 1). One patient had prostate cancer of neuroendocrine differentiation. In two patients, the prostate cancer diagnosis was made through biopsy of a distant metastasis, precluding Gleason scoring.

WB DWI identified additional lesions not identified on the PSMA PET in 24% (n = 10) of patients. The most common sites of mismatched disease were lymph nodes (n = 6), followed by bones (n = 4), lung (n = 1), and liver (n = 1) in 14%, 10%, 2%, and 2% of patients, respectively. In the six patients that received ^18^F-DCFPyL, 17% (n = 1) of them had nodal mismatched disease, and in the 35 patients that received ^68^Ga-PSMA-11, 14% (n = 5) of them had nodal mismatched disease.

**Figure 1:**
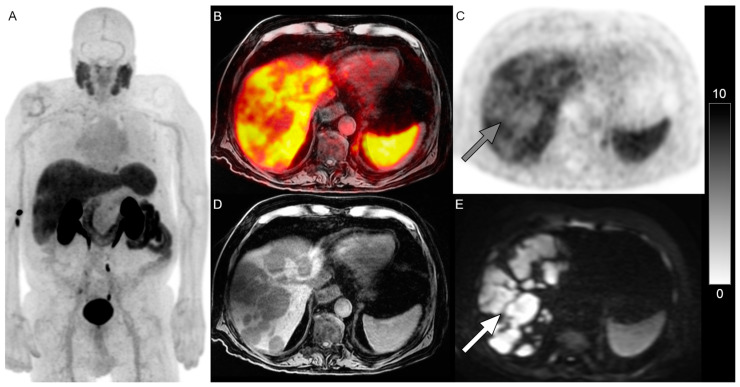
PET/MRI in a 78-year-old man with mCRPC with disease progression on cabazitaxel. Whole-body ^18^F-DCFPyL PSMA PET maximum-intensity projection image (**A**) shown. Extensive PSMA-negative hepatic metastases ((**B**), fused image, (**C**), gray arrow, PSMA PET) are identified on T1-weighted image (**D**) and b = 800 DWI ((**E**), white arrow). The scale bar of PET activity (SUVs) is on the right. Given the extent of PSMA-negative disease, RLT was not considered suitable. He died two months later.

**Figure 2:**
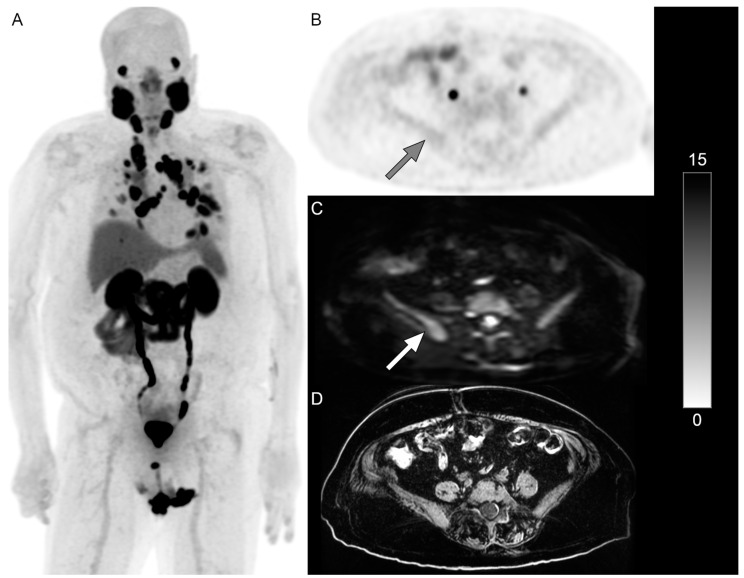
PET/MRI in a 78-year-old man with mCRPC with extensive mediastinal and supraclavicular nodal metastases ((**A**), whole-body ^18^F-DCFPyL PSMA PET maximum-intensity projection image). Only minimal PSMA activity was identified in the pelvic bones ((**B**), gray arrow); however, osseous metastases were identified on b = 800 DWI ((**C**), white arrow). A T1-weighted image (**D**) is also shown. The scale bar of PET activity (SUVs) is on the right. RLT was planned, but he died a month later prior to receiving RLT.

Excluding lymph nodes, DWI MRI identified additional lesions not identified on the PSMA PET in 14% (n = 6) of patients. It was noted that while 46% of patients in this analysis received RLT, none of these six patients with non-nodal mismatched disease received RLT. Figure 1 and Figure 2 show examples of patients with PSMA-negative disease that were identified on DWI.

**Figure 3:**
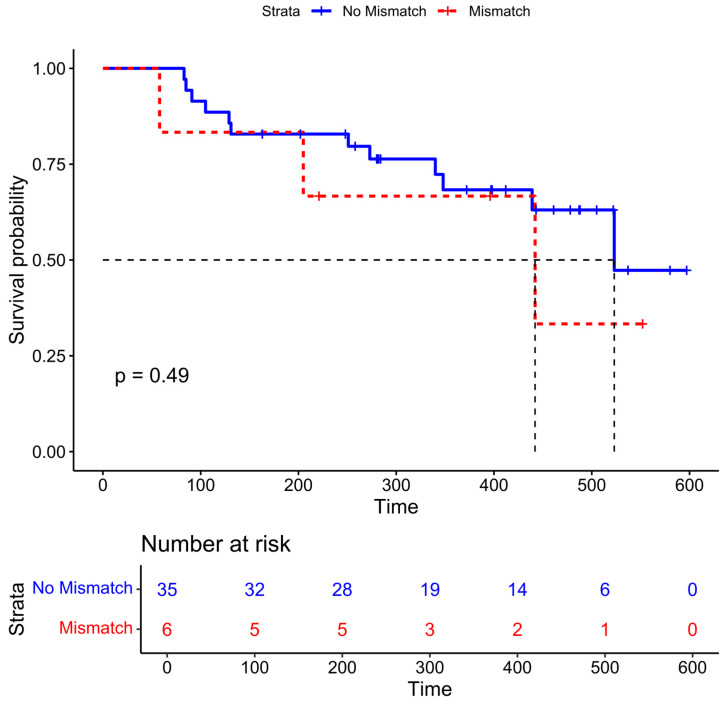
Kaplan–Meier survival curves for patients with non-nodal mismatched PSMA-negative disease (n = 6, red) and without (n = 35, blue). The *p*-value is from the log-rank test.

The median time to death or last follow-up was 348 days (IQR 221–461 days, range: 58–597 days). Six patients had non-nodal PSMA-negative disease that was identified on DWI. The median overall survival time was 442 days in those with mismatched disease compared to 523 days in those without, although this difference was not statistically significant (Figure 3, *p* = 0.49).

Both PSMA and DWI had good inter-reader agreement for categorizing the presence or absence of disease (ICCs and IQRs of 0.87 [0.84–0.89] and 0.72 [0.67–0.75], respectively), although agreement was significantly higher for PSMA PET compared to WB DWI (*p* < 0.001). The agreement of DWI improved to 0.85 (0.82–0.87) when only non-lymph node anatomic compartments were considered (Table 2).

## 4. Discussion

In this retrospective analysis of PSMA PET/MRIs at our institution, we found that WB DWI detected PSMA-negative lesions in 24% of patients, which could impact therapeutic decisions. PSMA PET had a higher inter-reader agreement compared to WB DWI, although both had good inter-reader agreements.

Since normal lymph nodes demonstrate restricted diffusion, the assessment of nodal disease on DWI is challenging and should be based on size alone. As such, we observed higher inter-reader agreement for DWI for non-nodal anatomic compartments. DWI is especially valuable for detecting liver metastases, where it has comparable performance to contrast-enhanced MRI, and it provides an incremental value when added to gadoxetic-acid-enhanced MRI, as the combination approach has the highest sensitivity [14,15]. Conversely, the physiological liver uptake of PSMA may obscure small lesions, especially when using ^18^F-DCFPyL compared to ^68^Ga-PSMA-11. The expected proportion of mismatched findings can depend on the background tracer activity and the radiopharmaceutical used.

Prior clinical trials of PSMA RLT described inferior outcomes in patients with PSMA-negative disease. In our cohort, the median overall survival time was 442 days in those with mismatched non-nodal disease compared to 523 days in those without. However, our study was not powered for this outcome, and the difference was not statistically significant. While further validation is needed before significant changes to patient management can be made based on DWI findings, we did note that none of the six patients with non-nodal PSMA-mismatched disease received RLT.

There has been an increasing effort toward standardization in the acquisition, interpretation, and reporting of WB MRI with the METastasis Reporting and Data System for Prostate Cancer (MET-RADS) [16]. WB DWI can be applied not only for the initial evaluation of patients with mCRPC but also for the assessment of treatment response, which is especially valuable in the setting of RLT.

Our study was limited by a small sample size and, thus, not powered for a statistical comparison of the overall survival, especially since only a minority of patients had non-nodal mismatched disease. This also precluded a meaningful evaluation of whether the proportions of mismatched disease differed between the ^68^Ga-PSMA-11 and ^18^F-DCFPyL PET. Another limitation was that the readers assessed the WB DWI and the PSMA PET images separately. This was necessary to determine the inter-reader agreement of each modality; however, this differs from routine clinical practice, where all images would be interpreted together, which may affect the generalizability. Finally, we did not have FDG PET as a reference standard to verify the reported mismatched disease that was detected on DWI.

In conclusion, we have demonstrated the feasibility of using WB DWI to identify PSMA-negative disease in patients undergoing PSMA PET. PSMA PET/MRI could be a practical approach to evaluating patients with mCRPC. Future studies in larger cohorts are needed to validate the diagnostic performance of WB DWI using FDG PET as a reference standard, compare it to PSMA PET to determine the proportion of mismatched disease, and stratify longitudinal outcomes based on DWI findings to support the usage of WB DWI in affecting patient management decisions.

## Figures and Tables

**Table 1:** Patient characteristics (n = 41). Median PSA and follow-up time with inter-quartile range and mean age with standard deviation were reported. For the categorical variables, proportions were reported.

Patient Characteristics		Result
Mean Age		71.9 ± 8.9 years
Median PSA		80.9 ng/mL (IQR: 40.6–487.0)
Median time since diagnosis to last follow-up/death		348 days (IQR: 221–461)
Castrate-resistant		100%
Received RLT		46%
Prior prostatectomy		24%
Prior definite radiation therapy		20%
	3 + 3	7%
	3 + 4	10%
	4 + 3	12%
	4 + 4	20%
Gleason score	4 + 5	20%
	5 + 4	12%
	5 + 5	12%
	Neuroendocrine	2%
	Not available	5%

**Table 2:** Inter-reader agreement for PSMA PET and WB DWI by anatomic compartment. Reader agreement was assessed using intra-class correlation coefficients. The 95% confidence intervals are shown in parentheses. * denotes anatomic compartments where the difference was statistically significant.

Compartment	DWI Agreement	PSMA Agreement
Pelvic nodes	0.87 (0.77–0.93)	0.91 (0.84–0.95)
RP nodes *	0.63 (0.41–0.79)	0.86 (0.75–0.92)
Mediastinal nodes *	0.28 (−0.03–0.54)	0.81 (0.67–0.89)
Inguinal nodes *	0.59 (0.35–0.76)	0.95 (0.90–0.97)
Supraclavicular nodes *	0.24 (−0.066–0.51)	0.74 (0.56–0.85)
Pelvic bone *	0.87 (0.78–0.93)	0.95 (0.90–0.97)
Lumbar spine *	0.78 (0.63–0.88)	0.96 (0.92–0.98)
Thoracic spine *	0.74 (0.57–0.85)	0.98 (0.97–0.99)
Cervical spine *	0.79 (0.63–0.88)	0.96 (0.93–0.98)
Ribs *	0.90 (0.82–0.95)	0.95 (0.91–0.97)
Upper extremities *	0.77 (0.61–0.87)	0.98 (0.96–0.99)
Lower extremities *	0.82 (0.70–0.90)	0.96 (0.92–0.98)
Lung *	0.35 (0.057–0.59)	0.87 (0.77–0.93)
Liver *	0.93 (0.87–0.96)	0.85 (0.74–0.92)
Other	0.31 (0.012–0.56)	0.43 (0.15–0.65)
All nodes *	0.57 (0.47–0.66)	0.85 (0.81–0.89)
Excluding nodes *	0.85 (0.82–0.87)	0.96 (0.95–0.96)
All bone *	0.81 (0.77–0.85)	0.96 (0.95–0.97)
Overall *	0.72 (0.67–0.75)	0.87 (0.84–0.89)

## Data Availability

Data generated or analyzed during the study are available from the corresponding author by request.

## Abbreviations

The following abbreviations are used in this manuscript:

WB DWI	Whole-body diffusion-weighted imaging
mCRPC	Metastatic castrate-resistant prostate cancer
